# Different Responses to Heat Shock Stress Revealed Heteromorphic Adaptation Strategy of *Pyropia haitanensis* (Bangiales, Rhodophyta)

**DOI:** 10.1371/journal.pone.0094354

**Published:** 2014-04-07

**Authors:** Qijun Luo, Zhenggang Zhu, Zhujun Zhu, Rui Yang, Feijian Qian, Haimin Chen, Xiaojun Yan

**Affiliations:** 1 Key Laboratory of Applied Marine Biotechnology, Ministry of Education, Ningbo, Zhejiang, China; 2 School of Marine Science, Ningbo University, Ningbo, Zhejiang, China; St. Georges University of London, United Kingdom

## Abstract

*Pyropia* has a unique heteromorphic life cycle with alternation stages between thallus and conchocelis, which lives at different water temperatures in different seasons. To better understand the different adaptation strategies for temperature stress, we tried to observe comparative biochemical changes of *Pyropia haitanensis* based on a short term heat shock model. The results showed that: (1) At normal temperature, free-living conchocelis contains significantly higher levels of H_2_O_2_, fatty acid-derived volatiles, the copy number of *Phrboh* and *Phhsp70* genes,the activities of NADPH oxidase and floridoside than those in thallus. The released H_2_O_2_ and NADPH oxidase activity of conchocelis were more than 7 times higher than those of thallus. The copy number of *Phrboh* in conchocelis was 32 times that in thallus. (2) After experiencing heat shock at 35°C for 30 min, the H_2_O_2_ contents, the mRNA levels of *Phrboh* and *Phhsp70*, NADPH oxidase activity and the floridoside content in thallus were all significantly increased. The mRNA levels of *Phrboh* increased 5.78 times in 5 min, NADPH oxidase activity increased 8.45 times in 20 min. (3) Whereas, in conchocelis, the changes in fatty acids and their down-stream volatiles predominated, significantly increasing levels of saturated fatty acids and decreasing levels of polyunsaturated fatty acids occurred, and the 8-carbon volatiles were accumulated. However, the changes in H_2_O_2_ content and expression of oxidant-related genes and enzymatic activity were not obvious. Overall, these results indicate that conchocelis maintains a high level of active protective apparatus to endure its survival at high temperature, while thallus exhibit typical stress responses to heat shock. It is concluded that *Pyropia haitanensis* has evolved a delicate strategy for temperature adaptation for its heteromorphic life cycle.

## Introduction

*Pyropia* (Bangiales, Rhodophyta), a marine red alga, has been cultivated for the past several centuries in Japan, Korea and China. Because of its economic importance, nutritional value and other health benefits, *Pyropia* cultivation has been expanded to other countries as well [1]. *Pyropia* grows on intertidal rocks and is extremely tolerant to the dynamic changes in environmental conditions, including the changes in temperature, desiccation, osmotic shock, and light intensity [2]. Therefore, *Pyropia* must have already developed a variety of highly effective strategies and mechanisms to overcome these environmental stresses.

High temperature is a situation that these algae always encounter. For example, *Pyropia haitanensis*, a typical algal species living in warm temperate zone, is cultured from September to March. Each year, there is a period from October to November, during which, the temperatures are increased. Because of heat stress, some of the algae are lost whereas other algae survive [3]. Thus, an interesting and important question one should ask is how can these algae overcome this heat stress? Zhang et al. suggested a physiological response pattern for *Pyropia* to high-temperature stimulation as follows: high temperature stress→ increased the levels of reactive oxygen species (ROS) →caused damage to cell membranes by ROS-mediated oxidation and increased malondialdehyde (MDA) levels →transmitted the signals of excessive ROS to the cells→ initially triggered antioxidation and osmoregulation systems to clean out the excessive ROS→ declined free radical levels [3]. The release of ROS and the damage to membranes of *Pyropia* caused by ROS have also been observed by several groups. The genes involved in the high-temperature tolerance of *Pyropia*, especially the genes encoding antioxidant enzymes, such as superoxide dismutase (SOD) [4], and heat shock proteins (Hsps) [5], have been investigated. These genes are similar to those of flowing plants [6]. In addition to possessing the thermo-tolerant mechanisms found in green plants, algae should also have some novel and unique mechanisms. Chemical deterrents have been regarded as the important defense strategies for marine algae. Many marine macro-algae produce a bouquet of halogenated organic compounds or oxylipins, such as prostaglandin, volatiles or non-volatile compounds. Some of them have been recognized as the defensive compounds [7]. For instance, the volatiles derived from oxylipin pathway of diatom are targeted against amphipod mesograzers [8]. At high temperature, cell membrane lipids will be oxidized, and the oxylipin pathway should be initiated. It has been well documented that the innate immunity of marine red algae involves eicosanoid oxylipin pathway. However, their biological roles in defending the abiotic stress still remain an enigma. On the other hand, to defend against the high-temperature stress, the low molecular weight sugars responsible for the osmotic regulation, the carbohydrate storage, and the cell wall synthesis will all be influenced [9]. Then, a key question to be asked is whether or not the changes in these low molecular weight sugars in red algae are related to the capability of tolerating high-temperature stress?

*Porphyra* has a unique heteromorphic life cycle with an alternation between a macroscopic foliose thallus which is the gametophytic stage, and a filamentous sporophyte stage called conchocelis. Conchocelis, considered as an independent organism, can survive under the adverse environmental conditions, since it is capable of withstanding the heat stress for the entire summer [10]. Therefore, it will be of scientific interest to study the different strategies that thallus and conchocelis use to deal with the heat short stress. In this study, we investigated and compared the thermo-tolerance capabilities of thallus and conchocelis of *P. haitanensis* from the aspects of both the expression of heat-related genes and the chemical strategies that they use for thermo-tolerance.

## Material and Methods

### Materials

The free-living conchocelis strain of *P. haitanensis*, HML, was obtained from the Marine Biotechnology Laboratory of Ningbo University and then cultured in cool-white fluorescent light at approximately 140-μmol photons m^−2^ s ^−1^ in a 12∶12 h light:dark (L:D) cycle at 20°C. The culture medium used was the filtered seawater containing 10 mg l^−1^ NO_3_^-^N and 0.4 mg l^−1^ PO _4_^3−^ -P, which was renewed every week.

The thallus of HML was cultured on a farm in Xiangshan County nearby Ningbo, Zhejiang province, China, where the longitude is 121.56.153 and the latitude is 29.05.065. The thallus was collected on Day 62 when the weather condition was fine and the temperature of seawater was 23.5°C. The length of thallus was 174.4±20.05 mm, the width was 4.16±0.86 mm, and the wet weight was 0.182 g thallus ^−1^. The thallus samples were dehydrated at room temperature before being stored at −20°C and were rehydrated with the filtered seawater at 20°C before use.

### Treatment of samples

The rehydrated thallus samples were brushed three times with filtered seawater and then sterilized by soaking in 0.7% KI solution for 10 min, thereby the unwanted algae and contaminants were removed. The thallus samples were preincubated in sterile seawater at 18°C for 24–48 h with cool-white fluorescent light at approximately 140-μmol photons m ^−2^ s ^−1^ in a 12∶12 h L: D cycle for 24–48 h.

The thallus and conchocelis were transferred to the preheated media and exposed to 35°C for 30 min. Thereafter, the samples were examined. The control samples were cultured in the same conditions without heat shock treatment (at 20°C).

### Quantification of H_2_O_2_ concentrations

The algae used in this experiment were grown in sterile seawater at a fresh-weight density of 7 mg ml^−1^. The mediums of the samples exposed to 35°C for different periods of time were collected for the detection of H_2_O_2_ by measuring the dimerization of (p-hydroxyphenyl) acetic acid (POHPAA) in the presence of horseradish peroxidase with a fluorometer. The POHPAA stock solution (6.13 μM POHPAA, 276.9 U L^−1^ POD, and 8.6 mM Tris-HCl, pH = 8.8) was added to the medium and incubated for 35 min in dark, the fluorescence was detected with wavelengths of excitation and emission of 313 and 400 nm, respectively. The H_2_O_2_ concentrations were calculated according to Miller's method [11].

### Analysis of fatty acids

Freeze-dried algal samples were extracted with CHCl_3_/CH_3_OH/H_2_O(1∶2∶0.8, v/v/v) to obtain total lipids, followed by saponification in MeOH-H_2_O (4∶1, v/v) with 5–6% KOH, under an N_2_ atmosphere at 60°C for 2 h. After cooling, the pH was adjusted below 1 with 1 M HCl. Successive extraction with hexane-chloroform (4∶1, v/v) three times yielded the total lipids. Fatty acid methyl esters were formed by heating with 14% BF_3_-CH_3_OH at 60°C for 1 h, which were then extracted with hexanechloroform (4∶1, v/v) twice and hexane once. Water (Milli-Q) was added to the combined extracts, the mixture was shaken and centrifuged at 3000 rpm for 15 min. The upper layer was separated and dehydrated, and the resulted product was treated with bis (trimethylsilyl) trifluoroacetamide (BSTFA) for 2 h at 60°C to derivate fatty acids to their trimethylsilyl ethers. The sample was then dried with N_2_, taken up in hexane and analyzed by QP2010 GC-MS (Shimadzu, Japan).

Gas chromatographic analysis was carried out using a SPB-50 fused silica capillary column, 30 m×0.25 mm×0.25 μm (Supelo, Bellefonte, PA, USA). The temperature of the injector was 250°C. Highly pure helium was used as the carrier gas with a column flow rate of 0.81 ml min^−1^ and a pre-column pressure of 73.0 kPa. After injection, the oven temperature was kept at 150°C for 3.5 min, and then programmed at a rate of 20°C min^−1^ to a temperature of 200°C and kept for 5 min, then programmed to a final temperature of 280°C at a rate of 5°C min^−1^, and kept for 30 min. The injection volume was 1 μl with split ratio of 50∶1. The mass spectrometer operated in electron compact mode with electron energy of 70 eV. The ion source temperature was set at 200°C, and the interface temperature was set at 250°C. The mass spectrometer scanned from m/z 50 to m/z 600.

### Analysis of volatile compounds

0.3 mg of treated and untreated thallus and conchocelis were ground in liquid nitrogen. A solid phase micro extraction (SPME) device (Supelco, Bellefonte, PA, USA) with a fiber coated with an absorbent phase made of polydimethylsiloxane/carboxen/divinylbenzene was used, and extraction was performed by headspace mode at 40°C for 50 min with magnetic stirring. After extraction, the SPME device was introduced in a GC splitless injector and maintained at 210°C for 5 min. Measurements were carried out using a Shimadzu QP2010 gas chromatography system fitted with a vocal column (60 m×0.32 mm, film thickness of 1.8 μm) (Supelco, Bellefonte, PA, USA) coupled with a Shimadzu QP2010 mass spectrometer (MS) (Shimadzu Scientific Instruments, Kyoto, Japan). Helium was used as the carrier gas. The oven temperature was programmed as the follows: 35°C for 3 min, then to 40°C at a rate of 3°C min^−1^ and held for 1 min, and finally increased to 21°C at a rate of 5°C min^−1^ and held for 25 min. The results were expressed as peak area. The mass spectra were obtained under electron ionization impact at 70 eV and data acquisition was performed over an m/z range of 45–1000. The analytes were identified on the basis of their retention time by comparing their mass spectra with those recorded in Nist 147 and Wiley 7 Spectrometry Library and those related to the previous analysis of pure references that are commercially available.

The volatile profiles acquired by the GC-MS measurements were analyzed by principal components analysis (PCA) using the SIMCA-P^+^ software package (V.12.0, Umetrics; Umea, Sweden).

### Real-time quantitative PCR analysis

Leafy thallus and conchocelis were used for the extraction of total RNA using AxyPre Multisource Total RNA Miniprep Kit (Axygen Bioscientific, Inc., Union City, CA, USA). All samples were ground into powder with liquid nitrogen and homogenized with cell lysis buffer, and all other steps followed the manufacturer's instructions. Total RNA was digested with DNaseI (TaKaRa Biotech Co., Dalian, China) for removal of genomic DNA residues. Single-stranded cDNA was synthesized from 1 μg of total RNA using Moloney murine leukaemia virus reverse transcriptase (Promega Biotech Co., Madison, WI, USA) and stored at −20°C.

The expression levels of *PhMn-sod*, *Phhsp70* and *Phrboh* genes were analyzed by qRT-PCR with a Stratagene Mx3005P real-time PCR system. The amplification of the *PhMn-sod*, *Phhsp70* and *Phrboh* cDNA fragments from *P. haitanensis* was undertaken using degenerate primers designed from the available sequences of *P. haitanensis* [GeneBank accession number EU715988 for *PhMn-sod*; number BQ631826 for *Phhsp*70; and number KF010298 for *Phrboh*, respectively]. Another pair of primers for 18S rRNA gene was used to amplify a 153 bp fragment of the 18S as the internal reference gene. The four pairs of primers used were as follows: *PhMn-sod-F* (5′-GCTGATGGAGGGCATTGTC-3′), *PhMn-sod-R* (5′-CGGTGTAGTTCTTGGCAATGA-3′) were used to amplify a *PhMn-sod* fragment of 158 bp; *Phhsp70-F* (5′-GTGGAGATGACTTTGACCAACA-3′) and *Phhsp70-R* (5′-GCTTGGGACCATCTTGTGTAG-3′) were used to amplify a *Phhsp70* fragment of 201 bp; *Phrboh-F*(5′-TGCCGCTCAAGACGACCTA-3′) and *Phrboh-R* (5′-CACCCACCACAGACCCAGA-3′) were used to amplify a *Phrboh* fragment of 90 bp; two *Ph18s* rRNA primers, and *Ph18s-F* (5′-AGTTAGGGGATCGAAGACGA-3′), *Ph18s-R* (5′-CAGCCTTGCGACCATACTC-3′), were used to amplify a 18s rRNA gene fragment as the internal control for qRT-PCR.

A total of 40 cycles of the qRT-PCR reactions were run as follows: 95°C for 15 s, 55°C for 20 s and 72°C for 10 s. Dissociation curves and no-cDNA controls were generated for each primer pair to detect nonspecific amplification. A standard curve was generated for each primer pair as well as for 18S, to which the gene expression levels were normalized by a comparative threshold cycle method. Finally, a ratio was calculated by comparing the normalized gene expression values in treated versus that in the untreated control for each sample.

### Measurement of enzyme activities

Control cultures or cultures (500 mg per sample) that had been exposed to 35°C for 30 min were ground into powder with liquid nitrogen and then homogenized on ice with 500 μl of pre-cooled lysis buffer containing protease inhibitors. The homogenate were centrifuged at 5000 rpm for 10 min at 4°C. The supernatant was used for detecting the activities of SOD and NADPH oxidase.

SOD activity was detected using a SOD detection kit (Nanjing Jiancheng Bioengineering Institute, Nanjing, China). NADPH oxidase activity was measured by a luminescence assay using a NADPH oxidase detection kit for plant (Genmed Scientifics. Wilmington, DE, U.S.A.). Manufacturer's instructions were followed.

### The quantitation of floridoside in *P. haitanensis*

The HPLC-MS analysis was carried out on Finnigan Surveyor and TSQ Quantum Access system (Thermo Fisher Scientific; Rockville, MD, USA) equipped with electrospray ionization (ESI) interfaced triple quadrupole mass spectrometer. In all cases, analyses were carried out at 25°C on Xbridge Amide (100 mm×3 mm, 3.5 μm, Waters), 10% 10 mM CH_3_COONH_4_ and 90% acetonitrile were used for isocratic elution. The flow rate was 0.3 ml min^−1^ and the injection volume was 10 μl. The HPLC-MS was operated in the positive ionization mode with data acquisition mode of SRM for quantification. The transitions monitored of the floridoside in SRM mode were m/z 253 to 119 (20eV) and m/z 253 to 89 (21eV). Ions at m/z 89 and 119 were utilized as quantitative ions.

### Statistical treatment of data

Data were analyzed by single factor ANOVA (α = 0.05) using SPSS (11.5). The difference between groups with a *P*<0.05 was regarded as statistically significant.

## Results

### The H_2_O_2_ released by *P. haitanensis* in responding to heat shock

**Figure 1:**
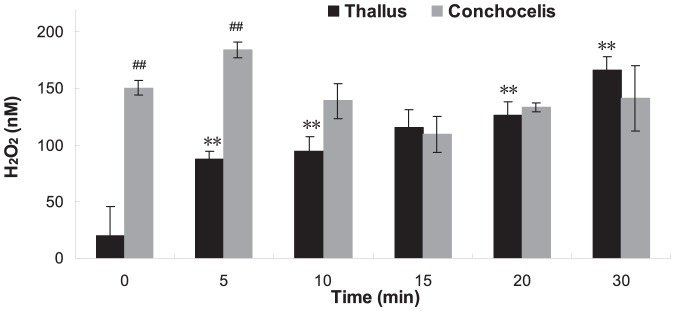
H_2_O_2_ concentrations in the medium of *P. haitanensis* during 30 min exposure to 35°C heat shock.

The H_2_O_2_ concentrations released from thallus and conchocelis cultured under normal temperature (20°C) were remarkably different (Figure 1). The initial H_2_O_2_ level of thallus without heat shock was low, but a high level of H_2_O_2_ was accumulated in conchocelis even without heat shock, which was 7.51 times the level of H_2_O_2_ released by thallus (*P*<0.01). Incubation of the thallus of *P. haitanensis* at 35°C resulted in a marked release of H_2_O_2_ within minutes. The stimulation of H_2_O_2_ release was time-dependent, with increasing the duration of heat shock, the algal response was increased and the maximal H_2_O_2_ concentration of about 166.5 nM was observed at 30 min. However, in conchocelis, the H_2_O_2_ level was kept high during the period of heat shock with some fluctuations. At 5 min, it was slightly increased, but from 10 min, it was decreased for a while and then returned to the normal level.

### The changes of fatty acids of thallus and conchocelis of *P. haitanensis* in responding to heat shock

Fatty acid compositions and the degree of fatty acid saturation under high temperature stress in both conchocelis and thallus of *P. haitanensis* were investigated. The number of fatty acids observed in conchocelis was greater than that observed in thallus regardless of treatments. The major fatty acids detected were 16∶0, 18∶0, 20∶4, and 20∶5 with additional fatty acids, 18∶4, 22∶5, and 22∶6, detected in conchocelis. Typically, the amounts of saturated fatty acids including 14∶0, 16∶0, 18∶0, 20∶0 detected in thallus were higher than those detected in conchocelis. Although fatty acid 20∶5 was in the greatest abundance in thallus, a larger amount of fatty acid 20∶4 was present in conchocelis. A short duration (30 min) of temperature increase above the normal growth temperature (from 20°C to 35°C) had an overall significantly greater effect on fatty acid compositions in conchocelis than in thallus. The amounts of saturated fatty acids, including 16∶0 and 18∶0, were increased significantly, but the amounts of polyunsaturated fatty acids, especially those of 20∶4 and 20∶5, were decreased significantly in conchocelis with the increase in temperature (Table 1) whereas thallus exhibited only minor differences in fatty acid levels between control and the 35°C treatments with a slight decrease of 20∶5 and an increase of 20∶4.

**Table 1:** The changes of fatty acids of thallus and conchocelis of *P. haitanensis* after heat shock (%).

Thallus			Conchocelis		
Fatty acids	Control without heat shock	After heat shock	Fatty acids	Control without heat shock	After heat shock
C14∶0	7.60±1.09	6.66±1.05	C14∶0	5.18±1.50	1.33±0.67
C16∶0	15.69±1.48	16.18±1.51	C16∶0	15.12±0.23	44.64±2.04^**^
C16∶1	6.22±0.75	6.74±0.77	C16∶1	6.55±0.97	4.15±0.99
C18∶0	10.80±1.57	9.22±1.08	C18∶0	7.17±1.59	29.24±1.42^**^
C18∶1	9.41±0.03	9.33±0.66	C18∶1	11.41±4.01	3.25±0.05*
C18∶2	5.92±0.59	6.11±0.12	C18∶2	6.12±1.23	2.69±0.71
C18∶3	0.87±0.32	0.82±0.67	C18∶3	0.10±0.09	-
C18∶4	-a	-	C18∶4	1.04±0.42	-
C20∶0	0.79±0.20	0.33±0.27	C20∶0	-	1.12±0.15
C20∶1	5.30±0.24	5.81±1.59	C20∶1	5.66±1.35	1.4±0.36
C20∶3	5.79±0.69	6.50±0.66	C20∶3	-	-
C20∶4	8.23±0.44	10.69±0.71	C20∶4	23.31±1.08	13.13±2.07^**^
C20∶5	23.36±2.15	21.61±3.00	C20∶5	17.60±2.58	9.74±1.33^**^
C22∶5	-	-	C22∶5	0.16±0.33	-
C22∶6	-	-	C22∶6	0.37±0.15	-

a “-” indicated undetected.

*p<0.05, **p<0.01 compared with control.

### The changes of volatiles of thallus and conchocelis of *P. haitanensis* in responding to heat shock

**Figure 2:**
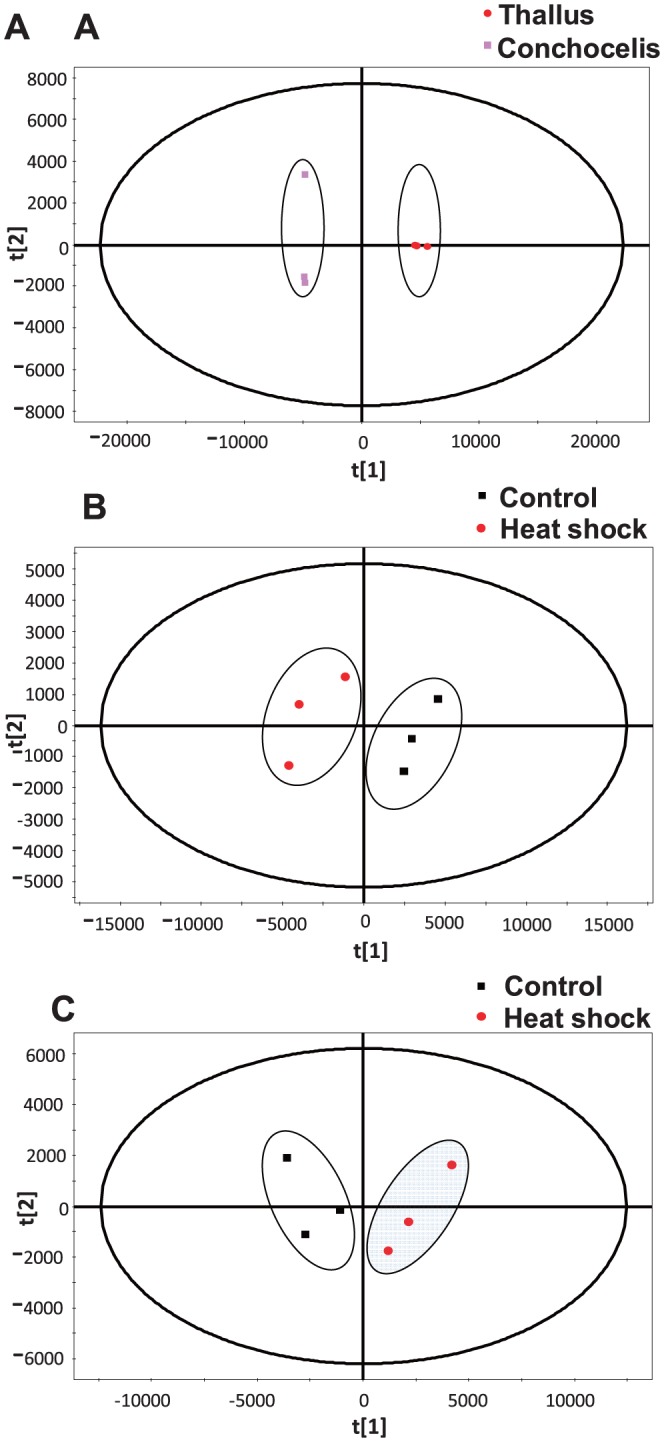
PCA scores scatter plot for the volatiles of *P. haitanensis in* responding to heat shock. A, PCA scores scatter plot for the volatiles of thallus and conchocelis of *P. haitanensis*. B and C represented PCA scores scatter plots for the volatiles of control and heat shocked thallus and conchocelis, respectively.

The different profiles of volatiles between thallus and conchocelis and the changes in volatiles after heat shock were analyzed by using PCA method, which provided a view on the similarities and dissimilarities between these samples. Figure 2A showed that two stages of *P. haitanensis* could be separated clearly, and the distance of the clusters between two stages was separated far away in the score plot, indicating an obvious difference in the profiles of volatiles between them. Heat shock induced the remarkable changes in volatiles in both thallus and conchocelis. In Figure 2B and 2C, the clusters of control group and the heat shock treated-group were separated clearly. We analyzed these volatiles in thallus and conchocelis before and after heat shock. The data were present in Table 2, which revealed that the volatiles in *P. haitanensis* were mainly consisted of aldehydes, alcohols and ketones. In addition, conchocelis also contained a fraction of alkene and alkane. In term of their compositions,no matter in thallus or in conchocelis, the 8-carbon structures such as 1-octanol, 2-octenal, 3-octaone, 1-octen-3-ol, 2-octen-1-ol, 3-octanol and etc were the dominant volatiles. However, the compositions of the 8-carbon volatiles were somewhat different between two groups. For example, 1-octen-3-ol, 2-octen-1-ol, 3-octanol were present in conchocelis but were absent in thallus whereas 2-octanal and octadienal were present in thallus but were absent in conchocelis. In term of the total contents of volatile, the total contents of volatiles in conchocelis were much higher than those in thallus, especially the total contents of 8-carbon volatiles. For instance, the content of 3-octaone in conchocelis was 2.75 times that in thallus and the contents of 1-octen-3-ol, 2-octen-1-ol were also very high in conchocelis. However, thallus contained a larger abundance of 8-heptadecene, which accounted for 1.14% of total volatiles. After heat shock, four new substances showed up in both thallus and conchocelis. Three 8-carbon volatiles, i.e. 4-octenoic acid, methyl ester, 2,3-octanedione, and 1-octanol showed up in thallus but their amounts were not very high. Among the four substances newly showed up in conchocelis, there was only one 8-carbon volatile, which was 4-octenoic acid but its content was relatively high, reaching 0.10 μg mg^-1^. In addition, the content of the newly generated 1-tetradecene was also very high. In term of the increased amounts of the already existing substances, there were only four substances whose amounts were increased, among which, the amounts of three 8-carbon volatiles (3-octanone, 2-methyl-, octatriene, 1,3-trans-5-trans-, and 3-octaone) were significantly increased. In conchocelis, there were 8 substances whose amounts were increased and, similarly, the amounts of several 8-carbon volatiles were significantly increased. For instance, the level of 3-octaone was increased by 6.78 times and the amounts of 3-octanol, 2-octenal, and 1-octen-3-ol were also highly increased. Especially for 1-octen-3-ol, its level was already very high before heat shock. After heat shock, its amount was further increased by 2.12 folds. Of courses, there were several substances whose levels were decreased after heat shock. In conchocelis, the substances whose levels were reduced were mainly the alkene and alkane substances, whereas in thallus, the levels of many types of aldehydes were reduced, particularly the crotonaldehyde substances such as 2-octenal, 2,4-octadienal and pentadecane.

**Table 2:** The changes of volatiles of thallus and conchocelis of *P. haitanensis* after heat shock (μg/100 mg).

Thallus			Conchocelis		
Volatiles	Control without heat shock	After heat shock	Volatiles	Control without heat shock	After heat shock
1-hexanol, 2-ethyl-	-a	2.69c1.79	1-tetradecene	-	11.46±10.17
4-octenoic acid, methyl ester	-	0.98±1.05	4-octenoic acid, methyl ester	-	10.07±7.70
2,3-octanedione	-	2.79±3.16	2,4-pentadienal	-	4.35±2.87
1-octanol	-	3.19±1.10	heptanal	-	3.94±3.94
3-octanone, 2-methyl-	1.17±0.23	11.35±2.30^**^	3-octaone	56.92±41.10	385.76±151.32^**^
octatriene, 1,3-trans-5-trans-	0.84±0.47	2.07±2.10*	benzaldehyde	4.55±2.92	23.68±13.01^**^
3-octaone	20.69±7.12	39.92±16.54*	hexanoic acid, methyl ester	1.29±0.27	5.84±2.37*
5-hepten-3-one,5-methyl-,sid/trans	9.95±4.02	11.38±6.68	nonanal	1.47±0.25	5.74±1.23*
hexadecanoic acid, methyl ester	36.02±33.01	28.84±9.63	3-octanol	9.55±10.05	36.68±24.93^**^
1-dodecen-3-yne	26.01±12.04	19.71±4.31	2-octenal	3.79±1.89	11.22±0.93^**^
8-heptadecene	1138.46±498.50	477.41±92.94*	1-octen-3-ol	56.30±46.31	119.47±40.70^**^
2-octanal, (E)-	2.14±0.31	0.87±0.66	2,5-diethylfuran	10.98±8.81	22.79±4.14
2,4-pentadienal	2.93±0.60	1.00±0.85	tetradecanoic acid, methyl ester	6.49±2.89	6.13±1.20
2-octenal	7.44±2.26	2.52±1.67*	1-tetradecanol	4.93±5.81	4.47±1.54
decenal	1.76±0.21	0.55±0.39	1-pentadecene	40.23±10.20	23.85±11.82
Trans,trans-2,4-octadienal	1.63±1.17	0.48±0.46	heptadecane	26.30±9.65	5.62±0.98^**^
pentadecane	16.64±9.28	4.03±0.62^**^	2-octen-1-ol	31.72±23.70	6.01±0.66^**^
1-tetradecanol	8.86±3.69	1.97±1.54*	8-heptadecene	19.97±10.25	-
nonanal	4.72±0.76	0.92±0.17*	1-dodecen-3-yne	28.53±12.40	-

a “-” indicated undetected.

*p<0.05, **p<0.01 compared with control.

### The differential gene expression patterns between conchocelis and thallus of *P. haitanensis* in responding to heat shock

**Figure 3:**
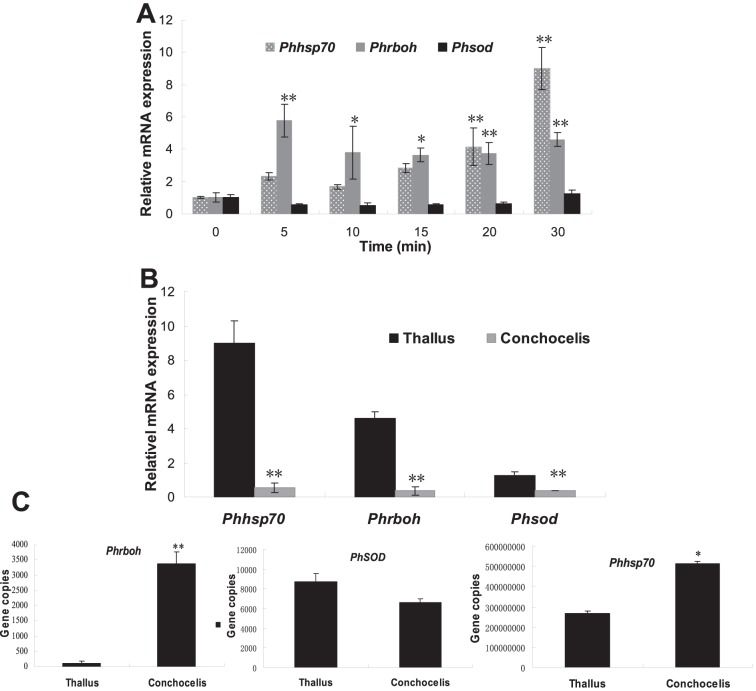
The gene expression of *P. haitanensis* in responding to heat shock. A, The gene expression response of thallus to different periods of heat shock. ^*^ *P*<0.05, ^**^ *P*<0.01 (n = 3) compared with that in control without heat shock. B, The different responses of thallus and conchocelis to heat shock for 30 min. The thallus or conchocelis of *P. haitanensis* was treated under 35°C. At different periods, the samples were collected, the mRNA was extracted and the expression levels of *PhMn-sod*, *Phhsp70* and *Phrboh* were analyzed by QRT-PCR. C, The gene copies of *PhMn-sod*, *Phhsp70* and *Phrboh* of thallus and conchocelis without heat shock. Data represented mean ± SD from three individual experiments. ^*^ *P*<0.05, ^**^ *P*<0.01 (n = 3) compared with those of the groups of thallus.

In this research, we chose three typical genes involved in responding to heat stress to investigate the activation of the cellular responses to heat stress. Hsp70 is a highly conserved protein which is expected to be induced under heat stress conditions [12]. Superoxide dismutase (SOD) and NADPH oxidase are assumed to be the key enzymes involved in the regulation of the balance between the generation and elimination of ROS caused by heat stress [13]. Figure 3A revealed that within 30 min of heat shock, the expression levels of *Phhsp70* and *Phrboh* were increased. Among which, the level of *Phhsp70* was increased with the increasing the duration of heat shock and reached maximal value in 30 min. *Phrboh* responded immediately to heat shock and its high expression occurred in 5 min. Its high expression level was kept thereafter. However, *Phsod* expression was down-regulated during the heat shock process and its expression level was reduced by near 2 folds from the 5^th^ to 15^th^ min after heat shock. In 20^th^ min, the reduction speed was slowed down but its expression was slightly up-regulated by 1.23 times in 30^th^ min. Comparing the differences in gene expression in responding to heat shock between thallus and conchocelis (Figure 3B), we found that within 30 min of heat shock, the expression levels of both *Phhsp70* and *Phrboh* in thallus were significantly increased whereas the expression levels of these genes in conchocelis were reduced. The expression of *Phhsp70* was down-regulated by 1.85 times and the expression of *Phrboh* and *Phsod* was down-regulated by 2.78 times. In addition, we applied the absolute quantitation to analyze the differences in gene copy number for several genes between thallus and conchocelis without heat shock (Figure 2C) and found that the copy number of *PhMn-sod* gene between thallus and conchocelis was not significantly different, but the copy number of *Phhsp70* in conchocelis was higher than that in thallus (*P*<0.05). However, the copy number of *Phrboh* gene was significantly different between conchocelis and thallus. It was very high in conchocelis, which was 32 times that in thallus (*P*<0.01).

### The different enzyme activity of *P. haitanensis* responded to heat shock

**Figure 4:**
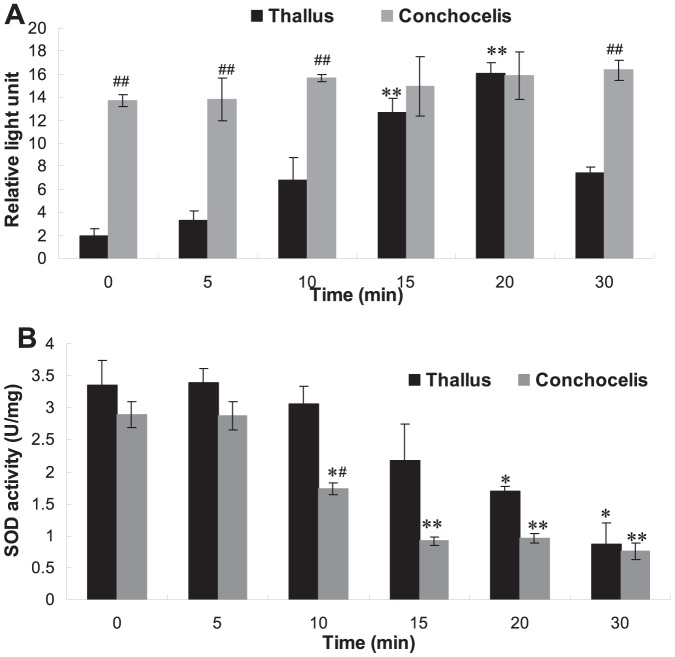
The enzyme activity of *P. haitanensis* in responding to heat shock. A, NADPH oxidase activities and B, SOD activities of thallus and conchocelis after heat shock at 35°C for 30 min. ^*^ *P*<0.05, ^**^ *P*<0.01 (n = 3) compared with those of control without heat shock. ^##^ *P*<0.01 (n = 3) compared with those of the groups of thallus.

Figure 4A showed that at the normal growth temperature, there were significant differences in the activities of NADPH oxidase between thallus and conchocelis, which were similar to the differences in the absolute gene copy number of the *Phrboh* gene encoding this enzyme between them. NADPH oxidase activity in conchocelis was much higher (7.18 times) than that in thallus. When being subjected to heat shock for a short period of time, NADPH oxidase activity in thallus was gradually and continuously increased and reached the maximal value in 20^th^ min, which was 8.45 times that without heat shock. Whereas NADPH oxidase activity was always kept at high levels in conchocelis but heat shock did not cause significant influence, and its enzymatic activity was not significantly increased. There was no big difference in SOD activity between thallus and conchocelis. When both thallus and conchocelis were subjected to heat shock at 35°C, SOD activities in both thallus and conchocelis were not changed within the first 5 min but were reduced thereafter, and reached the lowest level at 30^th^ min (Figure 4B).

### The floridoside response of *P. haitanensis* to heat shock

**Figure 5:**
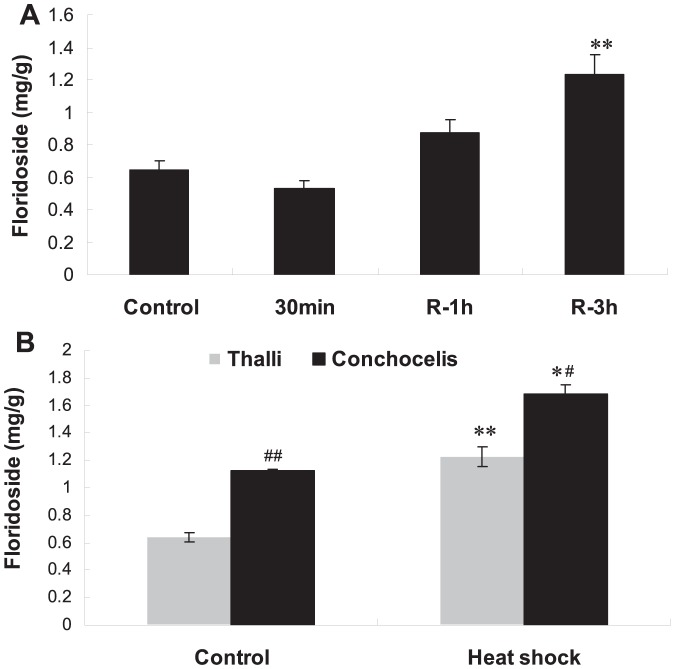
The changes of floridoside of *P. haitanensis* in responding to heat shock. A, The changes of floridoside of thallus treated at 35°C for different periods. ^**^ *P*<0.01 (n = 3) compared with those of control without heat shock. B, The different floridoside responses of thallus and conchocelis to heat shock after recovery for 3 h. The thallus or conchocelis of *P. haitanensis* was treated at 35°C for 30 min. The samples were then returned to 20°C and examined after the periods of 1 h and 3 h. The control samples were cultured under the same conditions without heat shock treatment. R-1 h and R-3 h indicated the samples returned to 20°C for 1 h and 3 h, respectively. ^*^ *P*<0.05, ^**^ *P*<0.01 (n = 3) compared with that of control without heat shock. ^#^ *P*<0.05, ^##^ *P*<0.01 (n = 3) compared with that of the groups of thallus.

As a major photosynthetic product in the members of most orders of Rhodophyta, the content of floridoside was also changed after heat shock (Figure 5). In thallus, at 30^th^ min after heat shock, the floridoside content was not increased but slightly decreased. Within 3 h of recovery after heat shock (marked as R-3h in Figure 5), the floridoside content in thallus was significantly increased with time. Comparing the floridoside contents between thallus and conchocelis, we found that floridoside content in conchocelis was significantly higher than that in thallus (*P*<0.01) without heat shock. After heat shock, floridoside contents in both thallus and conchocelis were significantly increased.

## Discussion

*Pyropia* has a unique dimorphic life cycle consisting of two generations, a leafy gametophyte and a filamentous sporophyte stage, whose morphologies are completely different. Gametophytic blade stage experiences both the winter and spring seasons. After fertilization, produced zygotospores grow naturally inside the shells to form the filamentous sporophyte called the conchocelis. The conchocelis resettles during the summer-months under adverse environmental conditions until the end of autumn. Under suitable conditions, it releases conchospores, and germinates to give rise to the young chimeric thallus [2]. Thus, conchocelis is the stage during which *Pyropia* is in the most tolerant state to high temperature. Therefore, to investigate the different strategies that *Pyropia* uses to respond to high temperature during two stages of its life cycle, thallus and conchocelis, is of great significance for a better understanding of its mechanisms of getting through the hot summer and the general thermo-tolerant mechanisms among different organisms. *P. haitanensis* is an endemic species naturally distributed only along the coasts of South China. It lives in the water temperature higher than that at which *P. yezoensis*, the specie widely cultivated in eastern Asia, live [14]. The conchocelis of *P. haitanensis* can experience the temperature of 30°C during summer season. Thus, the studies using *P. haitanensis* as the investigated subject could be reflective for the values of our studies.

Based on the analyses of the difference in the compositions of fatty acids between thallus and conchocelis, we found that the contents of the fatty acids 16∶0, 20∶4 and 20∶5 were very high, which were similar to those of other red algae [15]. In term of their compositions, there were significant differences in the compositions of certain fatty acids between two generations. Conchocelis contained fewer saturated fatty acids but more polyunsaturated fatty acids. A general role states that organisms survive in low temperatures tend to produce more unsaturated fatty acids [16]. While there have been reports indicating that in the plants being stressed by high temperature, the contents of unsaturated fatty acids in their cell membranes were significantly increased. Several studies have indicated that the higher the contents of unsaturated fatty acids in cell membranes are, the less easily for plants to suffer the damage caused by high temperature stress, and also the stronger their thermo-tolerance is [17]. Thus, the contents of unsaturated fatty acids in cell membranes could be used as one of the indicators for investigating plant's thermo-tolerance. The higher contents of unsaturated fatty acids in cell membranes of conchocelis could be related to fact that conchocelis lives in summer when the water temperature is relatively high and it gradually produces the fatty acids with higher degree of unsaturation to adapt the changes brought about by the high temperature.

Environmental conditions can affect lipid compositions in organisms. Maintenance of membrane integrity in organisms represents a central mechanism for temperature tolerance. In responding to the high temperature, polyunsaturated fatty acids in cell membranes will be decreased and saturated fatty acids will be increased [18]. Such that, Rousch et al. monitored the changes in fatty acid profiles of marine diatoms during a high temperature stress, and indicated that the degree of fatty acid saturation was affected. The polyunsaturated fatty acids were decreased whereas the saturated fatty acids were increased. However, the duration of high temperature treatment in their experiments was relatively long. They took the 2 h-treatment as the short duration treatment and the 24 h-treatment as the long duration treatment but the difference in changes in polyunsaturated fatty acids that they observed was small. For instance, under the heat shock at 40°C for 2 h, polyunsaturated fatty acids in *Phaeodactylum tricornutum* were reduced to 49% from 55% at the temperature of 24°C [19]. Zhang et al. also observed that after high temperature stress, the membrane lipids of *P. haitanensis* were oxidatively damaged, the saturated fatty acids were increased whereas the polyunsaturated fatty acids were decreased. However, the duration of their high temperature treatment was also very long, i.e. 2–10 days [3]. Wang et al. treated *P. haitanensis* with temperature at 35°C for 1.5 h. They observed a slight change in the saturated and monounsaturated fatty acids, whereas the polyunsatuarated fatty acids were not decreased but slightly increased [20]. In our experiments, almost no changes in fatty acids in thallus were detected in a 30 min high temperature treatment. Thus, a long duration treatment may be required to cause the changes in fatty acids or the changes may be displayed during the subsequent recovery period. However, the changes in fatty acids in conchocelis of *P. haitanensis* after heat shock were surprising. After heat shock for 30 min, the phenomena of significantly increasing saturated fatty acids and significantly decreasing polyunsaturated fatty acids occurred. This changing trend is consistent with the responsive patterns of other organisms to a long-duration of heat shock [18]. Among which, a decrease in polyunsaturated fatty acids could be the consequence of the oxidation of these fatty acids in the membrane lipids. A part of the polyunsaturated fatty acids may be converted to volatiles via oxylipin pathway. Hydroperoxides are the initial products of lipid oxidation [21]. They could be metabolized into short-chain aldehydes or alcohols. In previous research, we have found that the oxylipins in *P. haitanensis* are different from those of other algae. No hydroperoxides were detected but an abundance of short-chain volatiles was found [22]. Thus, in this study, we investigated the changes in short-chain volatiles under high temperature stress between two generations of *P. haitanensis*. In both the conchocelis and thallus, the 8-carbon structures are the dominant volatiles. The precursors of these 8-carbon volatiles such as 2-octen-1-ol, 1-octen-3-ol and etc should be derived mainly from C20∶4 and C20∶5, respectively, in *P. haitanensis*. It is consist with the previous report that the evolutionarily ancient marine red algae are rich in various classes of eicosanoids [7]. After high temperature stress, there were significant differences in the changes of fatty acids and short-chain volatiles between two generations. In particularly, the total VOC contents, especially the 8-carbon structures, in conchocelis were higher than those in thallus. This should be directly related to the significant decrease in the C-20 fatty acids after heat shock. At the present, it is not clear what the roles of these increased volatiles play under the heat shock stress as many physiological functions of these volatiles have not been reported yet. Previous research has indicated that 1-octen-3-ol can be an antimicrobial compound and that it can influence different developmental processes during the life cycle of *Penicillium paneum* [23]. (*E*)-2-nonenal was reported to have a strong inhibitory effect on the plant pathogenic fungus *Botrytis cinerea* [24]. The use of (*E*)-2-nonenal to nondormant tubers could terminate sprout growth and prevent regrowth for 2–3 month [25]. Because increasing temperature and shortening the duration of light radiation can cause the development of conchocelis of *P. haitanensis* to conchospprangial filaments [26], the volatiles produced by conchocelis during temperature changes could be related to its developmental changes or related to its disease resistance under stress conditions or act as the secondary messengers. Regardless, these changes of volatiles in conchocelis at least indicate that in red algae, the changes in membrane lipids in conchocelis are triggered rapid response to adapt the high-temperature stress. This difference between thallus and conchocelis suggests that the processes governing the initiation of stress response and changes in lipids may be distinct in conchocelis stage.

In term of the transcriptional levels of several genes, the changes in transcriptional expression of these genes are consistent with those in higher plants and other algae, i.e. the expression of the heat shock-related genes is up-regulated. For example, we observed that the expression of *Phhsp70* was gradually increased with time while *Phrboh* rapidly responded to heat shock and its expression was increased within 5 min. NADPH oxidase is involved in the generation of the superoxide anions O_2_^-^, which is subsequently dismutated by SOD to H_2_O_2_. It has been shown that when under heat shock, the first biological response is to increase the cellular ROS levels. Zhang et al. also observed that under heat shock, *P. haitanensis* initiated the similar responses [3]. However, we found that the mRNA level of *PhMn-sod* was not increased but decreased within 30 min of heat shock. This observation is consistent with the reported decrease in the activities of anti-oxidant enzymes such as SOD and POD in a large majority of algae during the initial period of high-temperature stress [27]. In this study, we used the high temperature of 35°C for a short-term heat shock of 30 min. Our results indicate that within this short duration of heat shock, the anti-oxidant enzyme system in algal cells has not been effectively triggered yet. In this way, the cellular ROS were kept at high levels during the short period of heat shock stress, which trigger the secondary messengers or play a role in killing the pathogenic bacteria. However, during the late stage of heat shock and the recovery period after heat shock, in order to prevent the cells from the damages caused by excess ROS, the anti-oxidant enzyme system is triggered to get rid of the excess ROS. Therefore, we observed that during 30 min of heat shock, the mRNA level of *PhMn-sod* was increased slightly, whereas Yang et al. reported that the expression of *PhMn-sod* was gradually increased during 3 h of the recovery period after heat shock [4]. These results are relayed and connected to our results and are also consistent with the observation reported by Zhang et al. that during the late stage of heat shock, the activities of antioxidant enzymes were greatly increased [3].

After having experienced the entire summer, conchocelis, the high temperature-tolerant period of the life cycle of *P. haitanensis*, should be more adaptive than thallus to high temperature. Furthermore, conchocelis is also regarded as the dormant stage of the life cycle of *P. haitanensis* and many of its physiological processes, such as photosynthesis, are in the inactive state. Thus, we could ask whether or not there exists the uniqueness for the high temperature-tolerant mechanisms in conchocelis? The conchocelis used in this study is a free-living conchocelis. The growth temperature in laboratory was 20°C. Therefore, a rapid temperature increase to approximately 35°C should be sufficient to cause heat shock stress. However, we observed that the strategies that conchocelis used to respond to high-temperature stress were totally different from those used by thallus and other organisms to respond to high temperature stress. The expression of three heat shock-related genes in conchocelis was not up-regulated but was all down-regulated under the heat shock stress, indicating that conchocelis probably does not initiate the active responses to heat shock but has become more inactive, making itself in a non-responsive state and even in an opposite responsive state. First, heat shock proteins are the chaperones and they are responsible for ensuring the correct folding of the macro-biomolecules in responding to the environmental stresses such as heat shock. In higher plants, the signature response to high-temperature stress is a reduction in the synthesis of the normal proteins, which is accompanied by an accelerated expression of the heat responsive genes including Hsps [12]. There are two possibilities for the fact that *Phhsp70* is not up-regulated in conchocelis under heat shock stress. The first possibility is that heat shock proteins are a large protein family and the other members of this family may be involved in responding to heat shock in conchocelis. For instance, Waters et al. observed that small heat shock proteins (smHSPs) were accumulated to high levels in response to heat stress. Their functions are likely to be critical for the survival and recovery from heat stress [28]. In addition, there may be the existence of other protein molecules that play the roles similar to those played by Hsps in conchocelis cells. The second possibility is that the changes in oxidant system and anti-oxidant system in conchocelis are very unique. We observed that the expression of both *Phrboh* and *PhMn-sod* genes was down-regulated by 2.77 folds after high-temperature stress, indicating that under heat shock, the expression of enzymes related to ROS formation is neither initiated and the expression of anti-oxidant enzymes is nor up-regulated in conchocelis. However, when we conducted absolute quantitation via RT-PCR analyses on several genes, we found that the copy number of the initial template of *Phrboh* gene in conchocelis was much higher than that in thallus, indicating that this gene has been in the highly expressed state in conchocelis. In detection of the activities of NADPH oxidase and SOD, we found that NADPH oxidase activity in conchocelis was significantly higher than that in thallus. However, after heat shock, the activity of NADPH oxidase in thallus was significantly increased whereas its activity in conchocelis was only weakly changed. In addition, in conducting the analyses on H_2_O_2_ production in both thallus and conchocelis, we observed that the amount of H_2_O_2_ produced in conchocelis was much higher than that produced in thallus even without heat shock. Together, these observations indicate that the oxidative system in conchocelis is very unique. Conchocelis may continuously produce high levels of ROS to adapt the environment of high water temperature through maintaining high expression of oxidant enzymes and high activities of these enzymes in their cells, playing the roles in defending against the invasion of the harmful organisms or in continuously activating the signal transduction to respond to high temperature but not simply the temporal increase after heat shock. However, after heat shock, due to high-temperature stress on the cells, if ROS production is further increased as in thallus or other organisms, ROS would cause substantial damage to cells. Thus, the expression of the oxidant enzyme, *Phrboh*, was down-regulated and the expression of *PhMn-sod* was simultaneously down-regulated to maintain ROS at certain levels. In addition, we also observed that the absolute copy of *Phhsp70* gene in conchocelis was higher than that in thallus. Asamizu et al. compared the mRNA expression profiles between two generations of *P. yezoensis*. They found that heat shock protein genes were significantly abundant in the sporophyte as compared with those in the gametophyte [29]. This conclusion is highly agreeable with our conclusion, suggesting that the heat shock protein genes expressed in the sporophyte may also play a crucial role in high-temperature tolerance.

However, does the high level of ROS in conchocelis itself cause any damage to conchocelis? And when conchocelis is under high-temperature stress, the expression of heat shock-related genes is down-regulated, then there must be other ways of anti-oxidation to protect the macro-molecules such as proteins and lipids from the damages caused by ROS and to play a role in protection of protein structures and functions. Hou et al. observed that under high-temperature stress, the contents of soluble sugars were increased in the conchocelis of *P. yezoensis*. They stated that soluble sugars in plant cells played a role in osmotic regulation and in protection for the structural stability of cell membranes [30]. The bioorganisms usually utilize a number of low molecular sugars to protect proteins and cellular membranes from inactivation or denaturation caused by a variety of stress conditions. For example, freezing- and heat-tolerance in many organisms such as yeast, higher plants, insects, etc. is related to the presence of trehalose [9]. Glucosylglycerol has been found to be a characteristic of moderately halotolerant cytnobacteria [31] and mannitol is responsible for the osmoreulation, storage and regeneration of reducing power in bacteria, algae and plants [32]. In red algae, the heteroside floridoside exert a similar function. The biological function of floridoside as an organic compound involved in osmotic acclimation has been investigated. Floridoside is also considered as the main photosynthetic and reserve product in *Rhodophyta* [33]. Li et al. have revealed that floridoside is a carbon precursor for the synthesis of cell wall polysaccharide in the red microalga *Porphyridium sp* [34]. In this study, we observed that the content of floridoside in conchocelis was higher than that in thallus. Cell wall is the rigid outermost cell layer and has the structures with protective functions. Zhou et al. observed that cell wall was gradually thickened with the growth of this free-living conchoceli [35]. This observation is similar to that made in this study. These observations indicate that during the summer season with a longer daylight and a higher light intensity when conchocelis grows, through carrying out photosynthesis, conchocelis can firstly synthesize a large amount of floridoside, a carbon precursor, which is then used to further synthesize the cell wall polysaccharides, enabling the cell wall to become thickened to defend against the invasion of extracellular harmful organisms, and to provide a stable internal environment for the synthesis of the intracellular substances and, at the same time, to control the loss of intracellular substances. This thickened cell wall is specifically for resisting the unfavorable environmental conditions. Furthermore, during the summer season, the vegetative algal filaments strongly carry out photosynthesis and convert carbohydrates into polysaccharides, which become the red algal starch, providing free-living conchocelis with sufficient energy and organic substances for growth and development [35]. Additionally, sugar alcohols in general exert multiple functions in metabolism, that is, in addition to their roles as the organic osmolytes and compatible solutes, they can also act as antioxidants. Mannitol functions as a strong antioxidant in higher plants owing to its ability to scavenge free oxygen radicals [36], it is reasonable to assume such an antioxidant role in red algae as well. Floridoside is an antioxidant. Li et al. revealed that floridoside possessed a significant antioxidant capacity and was a potential inhibitor of matrix metalloproteinases-2(MMP-2) and MMP-9 [37]. Thus, in red algae, floridoside certainly plays similar physiological roles. In our study, we observed that an increase in floridoside occurred under high-temperature stress, suggesting that floridoside acts as an antioxidant and a compatible solute in *P. haitanensis* to protect the functions of proteins and lipids, and then, to reduce cytoplasmic and membrane damage. The high contents of floridoside in conchocelis and the increased floridoside content under high-temperature stress can explain the fact that this stage of *P. haitanensis* is capable of growing normally under high-temperature stress, i.e. through increasing the levels of antioxidants, it can avoid the damage to macro-biomolecules caused by excess ROS.

In summary, thallus and conchocelis represent two distinct stages during life cycle of *P. haitanensis* and their growth temperatures are quite different. In addition to their differences in morphology and chromosomal ploidy, they also totally differ in their physiological response to high temperature stress and the ways that they use to respond to these stresses. Through maintaining the high transcriptional level of *Pharaoh* and the continuing the high levels of enzymatic activities, conchocelis maintains high levels of ROS, which enable it to adaptively respond to the influences by the unfavorable factors under a high temperature environment during the summer season. At the same time, it maintains the oxidant-antioxidant balance through the relatively high level of floridoside and the high expression of heat shock protein to protect the macro-biomolecules from damage caused by high-temperature stress. When it is artificially stressed by high-temperature, conchocelis makes adaptive responses by rapidly and strongly changing fatty acids and their down-stream volatiles. For thallus, it uses the strategies that are commonly used by many bio-organisms to respond to the stresses, such as H_2_O_2_ burst, increased floridoside contents and up-regulation of heat shock-related genes.
